# Hospital-based health technology assessment of a screening rapid test MTBI (GFAP and UCH-L1 blood biomarkers) for mild traumatic brain injury

**DOI:** 10.1017/S026646232400477X

**Published:** 2024-12-10

**Authors:** Miriam Menacho Román, Jose Roberto Penedo Alonso, Audrey Morales Rodríguez, Inés Pecharromán de las Heras, Agustina Vicente Bartulos, Ignacio Arribas Gómez, Nieves Plana Farrás

**Affiliations:** 1 Clinical Biochemistry Department. University Hospital Ramon y Cajal, Madrid, Spain; 2 Emergency Department. University Hospital Ramon y Cajal, Madrid, Spain; 3 Radiodiagnostic Department. University Hospital Ramon y Cajal, Madrid, Spain; 4 Biostatistics Unit. University Hospital Ramon y Cajal, Madrid, Spain

**Keywords:** traumatic brain injury, biomarkers, GFAP and UCHL1

## Abstract

**Background:**

The assessment of technology in hospital settings is a crucial step towards ensuring the delivery of efficient, effective, and safe healthcare.

**Objective:**

This study conducts a Hospital-Based Health Technology Assessment to evaluate the efficacy of a screening rapid test for mild Traumatic Brain Injury (mild TBI) utilizing blood biomarkers, specifically Glial Fibrillary Acidic Protein (GFAP) and Ubiquitin C-terminal Hydrolase L1 (UCH-L1). The assessment focuses on the clinical utility and performance characteristics of the proposed rapid test within a hospital setting.

**Methods:**

The screening model was meticulously examined for its ability to accurately detect mild TBI, considering the sensitivity and specificity of GFAP and UCH-L1 as blood biomarkers. The study involved a thorough evaluation of the test’s diagnostic accuracy, comparing its outcomes with established standards for mild TBI diagnosis.

Results from the Hospital-Based Health Technology Assessment highlight the potential of the GFAP and UCH-L1 blood biomarker-based rapid test as an efficient screening tool for mild TBI within a hospital environment. The evidence results show that the test is highly sensitive (91 percent to 100 percent) for the prediction of acute traumatic intracranial lesions, which helps rule out injury when the result is negative. When used within 12 hours of injury in adult patients with mild TBI, this test holds promise in reducing the utilization of CT.

**Conclusion:**

The findings contribute valuable insights into the feasibility and reliability of implementing this technology for timely and accurate identification of mild TBI, enhancing clinical decision making and patient care in hospital settings.

## Introduction

The assessment of technology in hospital settings is a crucial step towards ensuring the delivery of efficient, effective, and safe healthcare. Despite the rapid advancement of technology, new tools, and devices are often introduced into clinical practice without undergoing a rigorous evaluation process. Therefore, assessing the safety and cost-effectiveness of these technologies is critical before widespread adoption. This paper aims to illustrate the process of evaluating a particular healthcare technology for screening mild traumatic brain injury (TBI) in our hospital.

Traumatic brain injury is one of the most important causes of death and disability worldwide (1;2). Mild TBI accounts for more than 80 percent of all TBI cases. It is more prevalent in adults over 75 years, children under five years, and adolescents or young adults between 15 and 24 years (3). In the care of TBI patients, it is crucial to rule out acute intracranial lesions (AIL) that may necessitate surgical intervention.

While there is agreement on using cranial computed tomography (CT) in patients with moderate or severe TBI, the routine use of brain CT in patients with mild TBI consumes many resources and generates unnecessary radiation exposure. Only a low percentage of mild TBI patients (approximately 1 percent) ultimately require neurosurgical intervention (4). Before recommending a CT to a patient with mild TBI, it is necessary to evaluate the presence of sure warning signs based on different clinical prediction rules such as the Canadian CT Head Rule (CCHR), American College of Emergency Physicians Clinical Policy (ACEP), New Orleans Criteria (NOC), or guidelines such as the National Institute for Health and Clinical Excellence (NICE). (5–7)

During the last decades, the diagnostic and prognostic value of various biomarkers of brain injury has increased, including tau protein, B-amyloid, neuron-specific enolase, neurofilaments, S100 beta, glial fibrillary acidic protein (GFAP), and carboxyterminal hydrolase L1 ubiquitin (UCH-L1) (8–11). GFAP is a protein derived from astrocyte tissue expressed and explicitly released in the brain during traumatic injury, ischemic events, and certain neurodegenerative disorders (12). GFAP is released into the blood following traumatic injury, with an early plasma peak on the first day (13). After this, it decreases progressively during the first week from the third day of evolution. Alternatively, UCH-L1 is one of the most abundant proteins in the brain. It is exclusively located in neurons and can be detected in the blood, with elevated serum levels early after head trauma (14). The primary reason for proposing the TBI biomarker in our hospital is its 12-hour window period, which is longer compared to other biomarkers that could be useful for the evaluation of patients with traumatic brain injury, such as S100B, which has a window period of only 3 hours.

Until the appearance of biomarkers, patients who went to the emergency room after suffering a TBI were initially cared for by nursing staff, who carried out a triage, called Manchester, in which they classified the patient as mild or moderate. These patients were attended by the emergency physician, who carried out an anamnesis to determine the lesion mechanism, how much time had passed since the TBI, the patient’s medical history, as well as any treatment he or she was undergoing. In addition, a physical examination was performed, which included a neurological examination to see the patient’s Glasgow Coma Scale (GCS). These patients usually have a mild TBI with a GCS score of 13–15. Laboratory tests were also performed in patients older than 65 years, anticoagulated, with antiplatelet therapy, with signs of intoxication by substances, and patients with coagulation disorders. Finally, a head CT scan was also performed.

In most patients, the CT result is normal, that is, without evidence of acute bleeding. A small percentage of them present some type of intracranial hemorrhage (subdural hematoma, intraparenchymal hemorrhage, and subarachnoid). These patients are evaluated by the neurosurgeon, who keeps the patient under observation for 24 hours to reassess neurologically. In the event that they present changes, another CT control is carried out, and if no changes are observed and there is no sign of worsening, they are discharged.

In 2022, the Food and Drug Administration approved a rapid test based on the combination of biomarkers GFAP and UCH-L1 (mTBI test) for use within the first 12 hours after mild-moderate TBI (15). The mTBI test is a chemiluminescent microparticle immunoassay test that measures the concentration of GFAP and UCH-L1 proteins in a plasma or serum sample with a total volume of 300 μL. The ALINITY i system is used to carry out this test. The interpretation of the mTBI test results is based on the cut-off values for GFAP and UCH-L1, which are 35 pg/mL and 400 pg/mL, respectively. If the concentrations of both biomarkers are below their respective cut-offs, the mTBI test reports a negative result. Conversely, if the concentration of one or both proteins is at or above its respective cut-off, the mTBI test reports a positive result. In our hospital, a total of 2888 cranial CTs were performed in 2021 and 3307 in 2022, which means that around 8–10, CTs are performed daily. It implies that in about 5–6 patients with traumatic brain injury CT would be avoided every day. In a review carried out in 2021 at our hospital a total of 1677 brain trauma patients who attended the emergency room of our hospital underwent a cranial CT in the last 6 months. Out of them, 223 patients (13 percent) had pathologic findings related to the trauma. We proposed that if the mTBI test result were negative, not perform a brain CT, while if the result were positive, the decision to perform a brain CT would depend on clinical judgment. The TBI test must have the advantage of having excellent sensitivity and an excellent capacity to rule out true negative cases.

Before being implemented as a laboratory test in the emergency department, we conducted a hospital-based health technology assessment based on published data to evaluate the accuracy and cost-effectiveness of the mTBI test as a screening tool for adult patients who tend to come to the emergency department room within the first 12 hours after a mild TBI.

## Methods

We utilized a GRADE Evidence to Decision (EtD) framework to guide our decision-making process (16). This framework provided a structured approach to facilitate the translation of evidence into clinical practice recommendations. EtD frameworks consist of three sections that reflect the main steps involved in going from evidence to a decision: formulating the question, assessing the evidence, and drawing conclusions (16).

We formulated our clinical question as follows: “In adult patients who present to the Emergency Department with suspected mild TBI (GCS score of 13–15), what is the diagnostic performance and of screening with blood mTBI test within the first 12 hours after injury to rule out acute intracranial lesions?.” In addition, we wanted to know what is the cost-effectiveness of such screening in the same setting. We described the details of the question, including the population, intervention, comparison, and outcomes (PICO) in Table 1. We adopted a hospital perspective.

**Table 1.** PICO

Technology	mTBI test based on biomarkers (GFAP and UCH-L1) concentrations in blood samples measured with the ALINITY i TBI analytical system
**Indication**	**Disease or clinical condition** Acute intracranial lesions (susceptible or not to neurosurgical treatment) **Target population** Adult patients ≥18 yr of age with suspected mild head injury (GCS score of 13–15) within 12 hr of injury and presenting with a loss of consciousness for less than 30 min. **Proposed use** Diagnosis
**Comparison**	Routine clinical practice (protocol in current use)
**Outcomes**	-Accuracy indices: sensitivity, specificity -Incremental cost per QALY gained -Avoided CT
**Perspective**	Medical management of the University Hospital Ramón y Cajal to help decision making on the indication of a health intervention
**Context**	University Hospital Ramón y Cajal (Madrid Health Service)

GCS: Glasgow Come Scale, CT: Computed tomography, and mTBI: combination of biomarkers GFAP and UCH-L1

We undertook a rapid review (17) to produce a knowledge synthesis promptly. We searched specialized databases, such as Trip Medical Database, the Canadian Agency for Drugs and Technologies in Health (CADTH), the NICE, the Spanish Network of Agencies for Assessing National Health System Technologies and Performance, Guiasalud, The National Health Service Economic Evaluation Database (NHS EED) and Cochrane Library, to identify related HTA reports, clinical practice guidelines, and systematic reviews. We also searched Medline (via Pubmed) to identify primary diagnostic accuracy studies. We included prospective or retrospective cohorts that assessed the diagnostic accuracy of the combined GFAP and UCH-L1 biomarkers in blood for acute intracranial lesions in mild TBI patients. We conducted an additional search in Medline specifically for cost-related studies.

We assessed the certainty of evidence for the outcomes: true positives (TP), true negatives (TN), false positives (FP), and false negatives (FN) using GRADE (18). The certainty of the evidence was rated as high, moderate, low, or very low considering the study design, risk of bias, consistency, indirectness, and publication bias domains. We presented the accuracy results in a summary of findings (SOF) table. Additionally, the criteria considered in the EtD framework included benefits and harms, the balance between benefits and harms, certainty of evidence, use of resources or costs, acceptability, and feasibility. We formulated a recommendation graded as strong or weak, either for or against.

## Results

**Figure 1.**
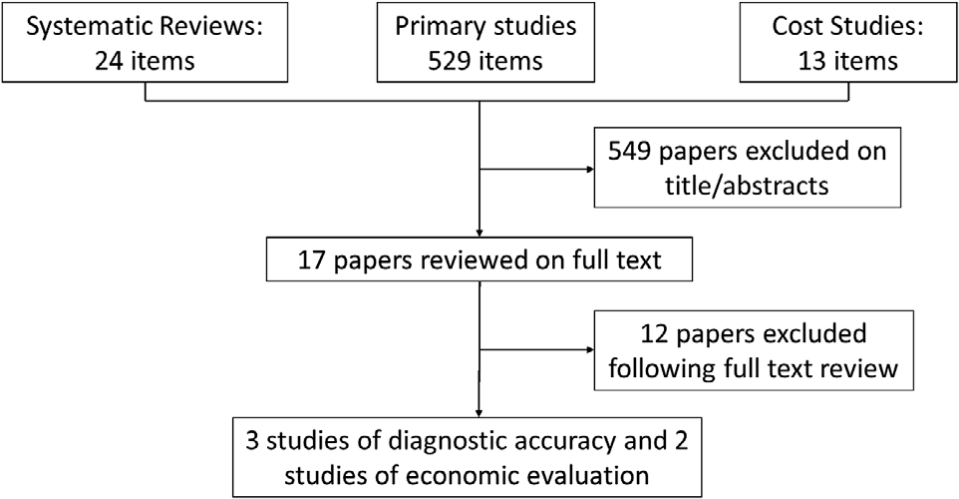
Study screening algorithm.

We searched for studies on 7th November 2022. We retrieved 566 references. After reading the abstracts, we excluded 549 references and selected 17 studies for further examination. We excluded 12 studies because they did not fulfill our inclusion criteria (Figure 1). The diagnostic accuracy of mTBI for detecting acute intracranial lesions in patients with mild TBI was assessed in three studies involving 2713 patients (19–21). Studies were published between 2021 and 2022. Two were prospective (16;17), and one was retrospective (19). The cut‐off values for the mTBI test varied between studies (Table 2). All studies compared mTBI with CT results.

**Table 2.** Summary of the characteristics and results of the primary diagnostic studies

First author, year, country	Study design	Population	N	Test (cut off)	Gold standard	Results (sensitivity and specificity)
Li, (19) 2022, USA	Retrospective Study period: December 2015–April 2017 Stanford HealthCare’s emergency department (ED)	Inclusion criteria: Adult patients ≥18 yr of age who had attended the Emergency Department with TBI and underwent a CT scan and blood sample were selected.	463	GFAP plus UCH-L1 Cut-off point: GFAP: 22 pg/mL UCH-L1: 327 pg/mL (cut-off points from the ALERT-TBI study (Bazarian 2018) (time since TBI not specified)	CT	Results in plasma GFAP plus UCH-L1 (total n = 463) Sensitivity: 100% Specificity: 11% NPV: 100% PPV: 29% Prevalence:122/463 (26.3%) 333 (71.9%) GCS 15 93 (20.1%) GCS 13–14 GCS 15 subgroup (n = 333): Sensitivity: 100% Specificity: 12% GCS 13–15 subgroup (n = 93): Sensitivity: 100% Specificity: 5%
Papa, (21) 2022, USA	Prospective Study period: 2010–2014 Premier Trauma Center	Adults ≥18 Mild TBI GCS: 13–15	349	GFAP plus UCH-L1 Test: Banyan Biomarkers (serum) Time: first 4 hr Cut-off point 1: GFAP: 67 pg/mL UCH-L1: 189 pg/mL Cut-off point 2: GFAP: 30 pg/mL UCH-L1: 327 pg/mL	CT (acute traumatic intracranial injury)	Cut-off point 1: Sensitivity: 100% (95%CI, 82–100) Specificity: 25% (95%CI, 20–30) Cut-off point 2: Sensitivity: 91% (95%CI, 70%–98%) Specificity: 20% (95%CI, 16%–24%) 90% GCS 15 Prevalence:23/349 (6.6%) CCHR and NOC sensitivity of 100% (95% CI, 82%–100%) and NEXUS II of 83% (95%CI, 60%–94%). NEXUS II had the highest specificity 52% (95%CI, 47%–58%), followed by CCHR 33% (95%CI, 28%–39%) and NOC with a specificity of 16% (95% CI, 12%–20%).
Bazarian, (20) 2021, USA and Europe	Prospective study period: 2012–2014 Secondary analysis of the ALERT-TBI study (Bazarian 2018) Multicenter (22 centers)	Adults ≥18 Mild TBI GCS: 9–15	2011 participated in the ALERT-TBI N = 1901 were analyzed (those with CT and GCS: 13–15)	GFAP plus UCH-L1 quick test (i-STAT TBI plasma) in the first 12 hr Cut point: GFAP: 30 pg/mL UCH-L1: 360 pg/mL	CT	Sensitivity:95.8% (95%CI 90.6–98.2) Specificity:40.4% (95%CI 38.2–42.7) GCS Subgroup 15: Sensitivity:95.7% (95%CI 89.6–98.3) Specificity:41.1% (95%CI 38.7–43.4) Prevalence:120/1901 (6.3%)

TBI: Trauma Brain Injury, GCS: Glasgow Come Scale, CT: Computed Tomography, and CI: Confidence Intervals

The prevalence of acute intracranial injury ranged from 6.3 percent (20) to 26.3 percent (19) between studies. The sensitivity of the mTBI test ranged from 91 percent to 100 percent (moderate certainty of evidence), and the specificity ranged from 11 percent to 41 percent (low certainty of evidence) (Table 3). Considering a prevalence of acute intracranial lesions of 6 percent, we found that 55–60 patients with mild TBI of 1000 would be correctly diagnosed with acute intracranial lesions and 103–386 of 1000 would be correctly diagnosed without acute intracranial lesions using the mTBI test. However, 554–837 of 1000 patients would be wrongly considered as having acute intracranial lesions (false positive), while 0–5 of 1000 patients might be incorrectly considered as not having acute intracranial lesions (false negative) (Table 3).

**Table 3.** Degree of certainty of the evidence. GRADE

Test	Number of participants (studies)	Accuracy Indeces	Test Result	Number of results per 1000 patients evaluated with the test (95%CI)			GRADE
				Prevalence (%)			
				6% (median)	10%	15%	
GFAP and UCHL1	2713 (3) (19–21)	Sensitivity (range): 91–100%	TP (patients correctly classified as having AIL)	55–60	91–100	137–150	⊕ ⊕ ⊕ ⭕ Moderate^a^
			FN (patients incorrectly classified as not having AIL)	0–5	0–9	0–13	⊕ ⊕ ⊕ ⭕ Moderate^a^
		Specificity (range): 11–41.1%	TN (patients correctly classified as not having AIL)	103–386	99–370	94–349	⊕ ⊕ ⭕ ⭕ Low^a^ ^,^^b^
			FP (patients incorrectly classified having AIL)	554–837	530–801	501–756	⊕ ⊕ ⭕ ⭕ Low^a^ ^,^^b^

Title: Rapid mTBI test based on blood biomarkers for managing patients with mild TBI (GFAP and UCH-L1).

Patients or population: Adult patients ≥18 yr of age with suspected mild head injury (GCS 13–15) within 12 hr of injury.

Context: Specialized Care.

Intervention: Combined determination of biomarkers in a blood sample (GFAP and UCH-L1) with the ALINITY i mTBI analytical system.

Comparison: Standard care.

Cut-off points: Li’s study (19) GFAP: 22 pg/mL and UCHL1: 327 pg/mL. Papa’s study (21) GFAP: 30 pg/mL and UCHL1: 327 pg/mL. Bazarian’s study (20): GFAP: 30 pg/mL and UCHL1 360 pg/mL

TP: True Negative, FP: False Positive, TN: True Negative, FN: False Negative. AIL: Acute Intracranial Lesions, and CI: Confidence Intervals. We illustrate possible clinical scenarios, by example, for a 10 percent prevalence of AIL, the GFAP and UCHL1 biomarkers accurately identify 91–100 patients per 1,000 as having AIL, with a sensitivity range of 91 percent to 100 percent, missing only 0–9 cases. Specificity, however, is lower (11 percent to 41.1 percent), meaning the test correctly excludes AIL in only 99–370 patients per 1000 while misclassifying 530–801 patients as positive.

a Downgraded one level for risk of bias of studies.

b Downgraded one level for inconsistency (high heterogeneity).

We found two studies (22;23) evaluating the cost-effectiveness of the mTBI test. A French study comparing the mTBI test with brain CT (22) concluded that patients assessed with the GFAP and UCH-L1 biomarker underwent fewer CT scans (677 vs. 1000). The patients had a similar number of emergency room visits and quality-adjusted life years (QALYs) gained. The cost savings were 4,150 euros per 1000 patients. A USA study (23) concluded that screening with mTBI would be cost-effective if the unit cost of the test were equal to or less than $308.96 with a prevalence of intracranial lesions in mild TBI of 10 percent. If the unit cost were higher than that, performing CT scans on all patients would be more cost-effective. The analysis in Su’s study (23) was performed at a WTP (willingness to pay) of 50,000 USD per QALY. The authors note that raising the WTP threshold to 100,000 USD per QALY (24) has only a minimal effect on the threshold cost of the biomarker screen (312.70 USD). In Spain, the cost-effectiveness threshold is not as clearly defined as in other countries; however, a threshold from 25,000–30,000 EUR per quality-adjusted life year (QALY) is commonly accepted. The cost of the test in Spain is 32 EUR, which is well below the thresholds applied in our country (25).

In 2022, a French guideline, “*Prise en charge des patients presentant un Traumatisme crânien leger de l’adulte*” (26), already recommends a combined blood test (UCH-L1 and GFAP) within 12 hours of mild TBI in patients classified as intermediate risk (*strongly recommendation*, GRADE). The guideline defined patients with intermediate-risk those aged 65 years or older on mono-antiplatelet therapy, those with a GCS lower than 15 at 2 hours after trauma with intoxication, those with high kinetic trauma, and those with amnesia for events occurring more than 30 minutes before the trauma.

We felt like a strong recommendation in favor of using the rapid test based on blood biomarkers (GFAP and UCH-L1) for the management of adult patients with mild TBI (GCS score of 13–15) within the first 12 hours post-injury was warranted based on the available evidence. We present in Table 4 a summary of the criteria and judgments considered in the EtD framework for making a decision.

**Table 4.**
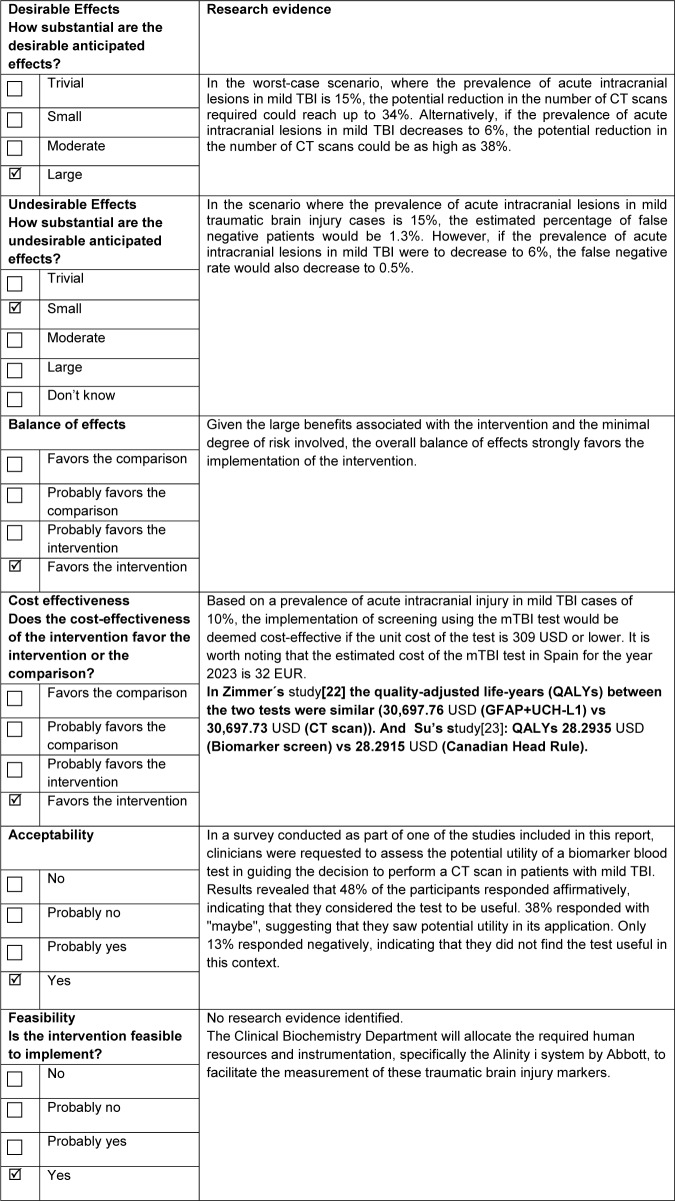
Criteria decision explanation

TBI: Trauma Brain Injury, CT: Computed Tomography, and mTBI: combination of biomarkers GFAP and UCH-L1

## Discussion

This article provides a review of the evidence regarding the diagnostic performance of the rapid mTBI test based on blood biomarkers (GFAP and UCH-L1) for the management of adult patients with mild TBI (GCS score of 13–15) within the first 12 hours post-injury, along with the assessment of this evidence to generate a GRADE-based recommendation.

The evidence results based on 3 studies (2713 participants) show that the test is highly sensitive (91 percent to 100 percent) but limited specificity (11 percent to 41 percent) for the prediction of acute traumatic intracranial lesions. A highly sensitive helps rule out injury when the result is negative. When used within 12 hours of injury in adult patients with mild TBI patients for whom a CT scan is deemed clinically necessary, this test holds promise in reducing the utilization of CT and, consequently, reducing radiation exposure. We think that this test has the potential to serve as a valuable decision-support tool in the context of the Emergency Department, aiding in the management of these patients. As a limitation of the evaluation, we recognize that the small number of included studies limits the generalisability of our findings. In addition, the variability in cut-off values between studies is a potential source of heterogeneity.

Although the decision to incorporate the biomarkers was made based on the evidence previously presented, in a recent update (August 2024), we have identified four new cohort accuracy studies (19;27–29). The information obtained in this new search has been included in the supplementary materials, and according to the results, it would not have affected the decision made.

We anticipated that there might be some resistance among physicians regarding the implementation of this test in the Emergency Department. This resistance can arise due to risk aversion, lack of familiarity or knowledge, economic concerns, and regulatory issues. Overcoming this resistance requires clear communication of benefits and scientific evidence, as well as proper training of medical personnel. By doing so, successful adoption of the new biomarker can be achieved, and clinical practice improved.

We are implementing a comprehensive plan that includes training and education. It is critical that the trained implementation plan is tailored to the specific needs and characteristics of the new biomarker and the clinical setting in which it will be implemented. This may include collaborating with subject matter experts, conducting hands-on workshops, developing educational materials, and ongoing follow-up to assess the effectiveness of the training and address any additional issues or resistance that may arise.

When a new biomarker is introduced, clinical practice guidelines can support its implementation in the following ways: evaluation of the evidence, recommendations for use, implementation framework, identification of target populations, and continuous updating.

In our hospital, the use of the mTBI biomarker was approved by the committee for new technologies for care practice, after various meetings had been held with the emergency service to publicize the management of mTBI, it has been enabled in the catalog of the Service of Biochemistry, being available for its realization 24–7.

We have been working with the biomarker for 11 months, it has been observed that little by little its request is increasing, although more time is needed for the first significant evaluation. The implementation started in March 2023. Although the test is proposed to be used sequentially in the first 6 months of implementation in the Emergency Department, what has been observed is a parallel use of the biomarker and CT in many cases, which has led to the results so far not being as optimistic as expected. We have analyzed the first 6 months after implementing the new biomarker: out of 955 adult brain trauma patients, 815 had positive TBI, 65 (8 percent) had CT with findings, 734 (90 percent) had CT without findings, and 16 (2 percent) CT was not performed. Of the 140 with negative TBI, 2 patients (1.4 percent) had CT with findings, 110 (78.6 percent) had CT without findings, and 28 (20 percent) had no CT performed. At the beginning of the implementation of the new test, we had the expectation of saving about 30–35 percent on CT scans. Later, taking into account our own results, we had the expectation of saving about 15 percent of CT scans, and this is the objective we actually have. We hope that with the experience accumulated the emergency clinicians get the necessary confidence on the test. Estimating costs for 1,000 patients, and 20 percent of them having mild brain trauma, we would save 4,000 EUR. For a prevalence of 15 percent, we would have an extracost of 5,000 EUR. Future research in our hospital will focus on assessing the long-term outcomes of patients screened with the mTBI test, as well as comparing the test’s performance across different patient populations.

## Supporting information

Menacho Román et al. supplementary material Menacho Román et al. supplementary material

## Supplementary material

The supplementary material for this article can be found at http://doi.org/10.1017/S026646232400477X.
