# Supplementary material for: Hospital-based health technology assessment of a screening rapid test MTBI (GFAP and UCH-L1 blood biomarkers) for mild traumatic brain injury
Source: Int J Technol Assess Health Care. 2024 Dec 10;41(1):e5. doi: 10.1017/S026646232400477X (PMC11703612; doi:10.1017/S026646232400477X)
Supplement: Menacho Román et al. supplementary material [file S026646232400477Xsup001.docx]

**SUPPLEMENTARY MATERIALS**

**Search Strategies**

Trip Medical Database (November 7^th^, 2022)

Search terms: "head injury" or "brain trauma"

CADTH Canadian Agency for Drugs and Technologies in Health (November 7^th^, 2022)

Search terms: "head injury" or "brain trauma"

NICE (November 7^th^, 2022)

Search terms: "head injury" or "brain trauma"

UpToDate (November 7^th^, 2022)

Search terms: "head injury" or "brain trauma"

GuíaSalud (November 7^th^, 2022)

Search terms: “traumatismo craneoencefalico”

Spanish Network of Agencies for the Assessment of Health Technologies and Benefits of the National Health System (November 7^th^, 2022)

Search terms: “traumatismo craneoencefalico”; “biomarcador”

MEDLINE (via PubMed)

Search Systematic reviews (November 7^th^, 2022)

("brain injuries, traumatic"[MeSH Terms] OR "brain trauma*"[Title/Abstract] OR "traumatic brain injur*"[Title/Abstract]) AND ("Glial Fibrillary Acidic Protein"[MeSH Terms] OR "Glial Fibrillary Acidic Protein"[Title/Abstract] OR "GFA-Protein"[Title/Abstract] OR "GFAP"[Title/Abstract] OR ("uchl1 protein human"[Supplementary Concept] OR "uchl1"[Title/Abstract] OR "blood biomarkers"[Title/Abstract]))

24 results

Search cost studies (November 7^th^, 2022):

("brain injuries, traumatic"[MeSH Terms] OR "brain trauma*"[Title/Abstract] OR "traumatic brain injur*"[Title/Abstract]) AND ("Glial Fibrillary Acidic Protein"[MeSH Terms] OR "Glial Fibrillary Acidic Protein"[Title/Abstract] OR "GFA-Protein"[Title/Abstract] OR "GFAP"[Title/Abstract] OR ("uchl1 protein human"[Supplementary Concept] OR "uchl1"[Title/Abstract] OR "blood biomarkers"[Title/Abstract]) AND ("cost"[Title/Abstract]))

13 results

Search for primary studies (November 15^th^, 2022)

("brain injuries, traumatic"[MeSH Terms] OR "brain trauma*"[Title/Abstract] OR "traumatic brain injur*"[Title/Abstract]) AND ("Glial Fibrillary Acidic Protein"[MeSH Terms] OR "Glial Fibrillary Acidic Protein"[Title/Abstract] OR "GFA-Protein"[Title/Abstract] OR "GFAP"[Title/Abstract] OR ("uchl1 protein human"[Supplementary Concept] OR "uchl1"[Title/Abstract] OR "blood biomarkers"[Title/Abstract]))

Filter: last five years

529 results

**New search** (August 2024)

Trivedi and colleagues[27] demonstrated the superiority of GFAP and UCH-L1 over S100B in predicting CT-positive intracranial lesions in mTBI patients within 6 hours of injury. When sampled within 9 to 12 hours of injury, the sensitivity and specificity of these biomarkers were 73 percent (95 percent CI 46-82 percent) and 83 percent (95 percent CI 57-91 percent), respectively. In Chayoua's study[28], the specificity of these biomarkers was lower: 19 percent (95 percent CI 14-25 percent), likely due to the eligibility criteria for head CT being based on the CHIP decision rule. Similarly, in Li's study[19], the specificity was very low (17 percent), with a sensitivity of 100 percent. Another study, including patients with a GCS score 14-15 and at least one associated risk factor—antiplatelet monotherapy, loss of consciousness, or posttraumatic amnesia for events 30 minutes before the injury—showed a sensitivity of 100 percent (95 percent CI 73.5-100 percent) and a specificity of 31.7 percent (95 percent CI 25.7-38.2 percent) for samples taken within 12 hours of injury[29]. All studies report an intracranial injury prevalence above 20 percent, except for the Oris 2023 study [29], which shows a prevalence more similar to our context at 5 percent.
